# The impact of perivascular adipose tissue characteristics on incident cardiovascular events in non-dialysis chronic kidney disease patients

**DOI:** 10.3389/fmed.2025.1547007

**Published:** 2025-06-20

**Authors:** Shaozhen Feng, Zhiman Lai, Miaorong Xue, Wenjiao Zhu, Qian Zhou, Xiaojie Ke, Xingdi Guo, Wei Chen, Shurong Li, Qunying Guo

**Affiliations:** ^1^Department of Nephrology, The First Affiliated Hospital of Sun Yat-sen University, Guangzhou, China; ^2^NHC Key Laboratory of Nephrology (Sun Yat-sen University) and Guangdong Provincial Key Laboratory of Nephrology, Guangzhou, China; ^3^Department of Radiology, The First Affiliated Hospital of Sun Yat-sen University, Guangzhou, China; ^4^Department of Medical Statistics, Clinical Trials Unit, The First Affiliated Hospital of Sun Yat-sen University, Guangzhou, China

**Keywords:** chronic kidney disease, epicardial adipose tissue, peri-coronary adipose tissue, thoracic peri-aortic adipose tissue, incident cardiovascular events

## Abstract

**Introduction:**

Patients with chronic kidney disease (CKD) face a high risk of developing cardiovascular disease. However, the relationship between perivascular adipose tissue characteristics and incident cardiovascular outcomes in non-dialysis patients with CKD remains unclear.

**Methods:**

This was prospective, observational, cohort study. A total of 220 CKD patients (stages G2–G5) without prior cardiovascular disease were enrolled. Attenuations and volumes of peri-coronary adipose tissue (PCAT), thoracic peri-aortic adipose tissue (TAT), and epicardial adipose tissue (EAT) were measured by ECG-gated non-enhanced computed tomography scans. Total coronary artery (CAC) and thoracic aorta calcification (TAC) were quantified with Agatston scores.

**Results:**

Lower estimated glomerular filtration rate (eGFR) was associated with higher attenuation of PCAT, TAT, and EAT, but volumes differences across eGFR tertile were not significant. Multivariate analysis indicated that age and body mass index (BMI) were independently associated with PCAT, TAT, and EAT volumes. Smoking and serum phosphorus levels correlated with PCAT attenuation, while proteinuria, BMI, HDL cholesterol, and white blood cell counts were linked to TAT attenuation. EAT attenuations was associated with age, serum creatinine, proteinuria, BMI, smoking, and systolic blood pressure. During a median follow-up of 26.56 months, 23 patients developed cardiovascular events, with high EAT volume (≥129.14 cm^3^) or TAT volume (≥36.38 cm^3^) correlating with increased events rates. Cox regression demonstrated EAT volume as an independent predictor of incident cardiovascular outcomes. A multivariable model showed EAT volume enhanced the Framingham risk score's predictive value, achieving an area under the curve (AUC) of 0.76 (95% CI 0.66–0.87).

**Conclusion:**

These findings suggest EAT volume significantly predicts incident cardiovascular events in non-dialysis CKD patients.

## Introduction

Chronic kidney disease (CKD) ranks as the seventh most common cause of death from non-communicable disease globally. Cardiovascular disease (CVD) is a major complication of CKD and the leading contributor to morbidity and mortality in CKD patients, surpassing the impact of kidney failure itself (1). Perivascular adipose tissues, specialized local adipose deposits surrounding blood vessels, exert localized pathological effects on the vasculature and plays significant roles in cardiovascular risk. Meta-analysis has demonstrated that perivascular adipose tissue was significantly correlated with cardiovascular risk factors, such as body mass index, hypertension, blood glucose, total cholesterol, triglycerides, low- and high- density lipoprotein cholesterol (2). However, further research is required to assess perivascular adipose tissue in patients with and without prior cardiovascular pathology.

Perivascular adipose tissue has been proved as a risk factor for cardiovascular outcomes, diabetes, and coronary artery disease, and tends to increases in the patients at risk of cardiovascular disease (3–5). Meta analysis also indicates that patients with CKD exhibit greater perivascular adipose tissue thickness and volume compared to controls without CKD (6). Despite these findings, information on the relationship between perivascular adipose tissue, particularly epicardial adipose tissue (EAT), peri-coronary adipose tissue (PCAT) and thoracic peri-aortic adipose tissue (TAT), and cardiovascular events occurrence in patients with CKD remains limited (7). Two studies involving advanced CKD or those on hemodialysis patients with CVD history suggested that EAT thickness can help predict adverse cardiovascular events (8, 9). While another study of non-dialyzed patients with CKD found that EAT's explanatory power for cardiovascular events was minimal after adjusting for traditional and uremia-related risk factors (10). Notably, all CKD patients in these studies had a history of cardiovascular disease. To the best of our knowledge, no previous study has explored the role of specific perivascular adipose tissue characteristics, such as volume and attenuation of PCAT, TAT, or EAT, in predicting incident cardiovascular outcomes in patients with non-dialysis CKD beyond traditional risk factors.

This study aims to (i) characterize the attenuation and volume of three types of perivascular adipose tissue, PCAT, TAT, and EAT, in non-dialysis patients with CKD and their associations with cardiovascular risk factors, and (ii) determine whether perivascular adipose tissue attenuation or volume can serve as clinical predictors of incident cardiovascular outcomes in patients with non-dialysis CKD.

## Materials and methods

### Study population

This study was prospective, observational, cohort study approved by the Medical Ethics Committee of the First Affiliated Hospital of Sun Yat-sen University (No. [2022]656). Written informed consent was obtained from all participants. A total of 247 patients with non-dialysis CKD were included, all of whom had readable ECG gated non-enhanced CT scans and had been admitted to the Department of Nephrology, the First Affiliated Hospital of Sun Yat-sen University, from May 1, 2022, to July 31, 2023.

CKD was defined by the presence of kidney damage or an estimated glomerular filtration rate (eGFR) lower than 60 ml/min/1.73 m^2^, persisting for at least 3 months, irrespective of the cause. eGFR was calculated using the simplified modification of diet in renal disease equation: eGFR [ml/min/1.73 m^2^] = 186 × Scr^−1.154^ × (age)^−0.203^ × (0.742, female) (11). Patients with an eGFR of < 90 ml/min/1.73 m^2^ (CKD stages G2–G5) and without CVD history were included. CVD was defined as a self-reported history of stroke, myocardial infarction, heart failure, transient ischemic attack, or coronary heart disease. Patients under 18 or over 80 years of age and those with acute kidney injury, chronic inflammatory disease, active malignancy, serologic positivity for human immunodeficiency virus, syphilis, viral hepatitis, or previous manifestations of coronary revascularization that could affect the measurement of vascular calcification were excluded (12).

**Figure 1:**
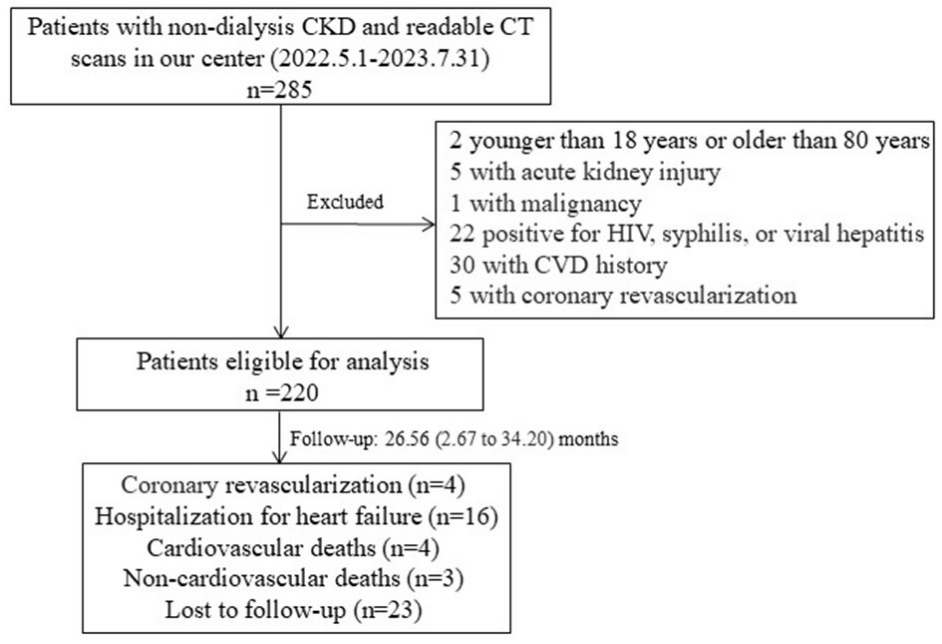
Flow chart of the participants in the study cohort. Two patients who had myocardial infarction were under coronary revascularization. CKD, chronic kidney disease; CT, computed tomography; HIV, human immunodeficiency virus.

A total of 220 patients were included in the analysis (Figure 1). Their clinical and demographic data were recorded. Body mass index (BMI) was calculated from their height and weight measurements. Diabetes was defined as treatment with oral hypoglycemic agents or insulin or fasting glucose levels of ≥7 mmol/L. Hypertension was defined by a diagnosis from a physician, a mean blood pressure of ≥140/90 mmHg, or the use of antihypertensive medication. Patients who were actively smoking at the time of enrollment or had smoked during the previous year were considered smokers. The use of lipid-lowering medications was also recorded.

### Cardiovascular adipose tissue measurement and quantification

CKD patients were scanned by ECG-gated non-enhanced CT using a third-generation dual-source CT scanner (Somatom Force; Siemens Healthineers, Forchheim, Germany). TAT, PCAT, and EAT were measured in each CT slice using semiautomatic software on a dedicated workstation (Coronary Analysis, Syngo. Via VB40; Siemens Healthineers). Adipose tissue was distinguished from other tissues using a threshold of −190 to −30 Hounsfield Units (HU).

TAT volume (cm^3^) was defined as the adipose tissue area surrounding the descending thoracic aorta (Supplementary Figure S1A). The superior edge of the aortic arch marked the proximal border, and the inferior border of the aortic hiatus of the diaphragm served as the distal border. The anterior borders included the left bronchus, esophagus, and interior border of the diaphragm crus. The posterior border corresponded to the anterior border of the vertebral foramen (13). TAT attenuation was calculated by recording the average attenuation (HU) of all voxels within this volume.

In the non-enhanced CT scans from patients with CKD, the image quality of the right coronary artery was more stable. In contrast, the proximal route of the left coronary artery was tortuous, making it difficult to distinguish it from the adjacent myocardium in some segments. Therefore, previously validated PCAT was quantified around the proximal right coronary artery (14). PCAT activity was measured 10–50 mm proximal to the right coronary artery ((Supplementary Figure S1B). PCAT volume (cm^3^) included three-dimensional concentric layers extending outward from the operator-traced vessel wall to a radial distance equivalent to the diameter of the target vessel from the outer border. The volume and attenuation of PCAT were quantified automatically.

EAT was defined as adipose tissue within the pericardium (Supplementary Figure S1C). The EAT boundaries were defined as follows: “1” for superior, pericardial reflection near the pulmonary artery and below the aortic arch; “2” for inferior, diaphragmatic transition; “3” for posterior, the line between the right and left main bronchi; “4” for anterior, the internal limit of the anterior thoracic wall (14). EAT volume (cm^3^) and mean attenuation (HU) were calculated automatically.

An operator with over 10 years of CT experience who was blinded to the clinical data (ZL) measured the perivascular adipose volume and attenuation. The volumes and attenuations of the TAT, PCAT, and EAT were also quantified in a randomly selected subset of participants (*n* = 25) for reproducibility analysis. The measurements were repeated at intervals of 3–4 weeks. Excellent intra-reader reproducibility was achieved for TAT, PCAT, and EAT volumes (correlation coefficients of 0.900, 0.892, and 0.915, respectively; *P* < 0.001) and TAT, PCAT, and EAT attenuations (correlation coefficients of 0.967, 0.928, and 0.926, respectively; *P* < 0.001).

### Measurement and quantification of coronary artery and thoracic aortic calcification

Calcification of the coronary arteries and the descending aorta was also measured using ECG-gated non-enhanced CT. The CT image acquisition range was from 2 cm above the aortic arch to the lower margin of the first lumbar vertebra. According to the patient's heart rate, the central phase of the cardiac cycle that triggered the scan was 30 or 70% of the R-R interval.

The extent of coronary artery calcification (CAC) and thoracic aorta calcification (TAC) was individually estimated as the Agatston score, quantified using semi-automated software on a dedicated workstation (CaScoring, Syngo. via VB40, Siemens Healthineers). Calcification was considered present at a threshold of 130 Hounsfield Units (HU) and a minimum area of 1.0 mm^2^ (15). CAC lesions were manually annotated and quantified for each of the four major coronary arteries by a single radiologist (SR. L) with over 10 years of experience in cardiovascular imaging. The scores were then totaled to calculate the total Agatston score.

### Follow-up and outcome data

We reviewed medical records and conducted telephone interviews to collect follow-up information regarding cardiovascular events until the end of August 2024 (Figure 1). Cardiovascular events were defined as death from cardiovascular causes or non-fatal myocardial infarction, ischemic stroke, hospitalization for unstable angina or heart failure, or coronary revascularization.

### Statistical analyses

Statistical analysis and image processing were performed using SPSS (IBM SPSS Inc, version 22.0) and GraphPad Prism 9.0. Continuous variables are presented as mean ± standard deviation or median (interquartile range) for non-normally distributed data. Normality was assessed via the Kolmogorov–Smirnov test. Categorical variables are presented as percentage (frequencies). All hypothesis tests were two-sided, with significance set at *P* < 0.05. For quantitative data, normal distribution variables were compared using independent-sample *t*-test (for two groups) or ANOVA (for multiple groups). Non-normally distributed variables were compared using the Mann–Whitney *U* test (for two groups) or the Kruskal–Wallis test (for multiple groups). Categorical variables were compared using chi-square tests or Fisher's exact probability tests.

Factors associated with PCAT, TAT, and EAT attenuation or volume were assessed using linear regression. Variables significant in univariate analysis (*P* < 0.05) or of clinical relevance were included in multivariate analysis using the enter method. Kaplan–Meier curves were used to estimated incident cardiovascular event rates based on PCAT volume (using the median of ≥3.37 vs. < 3.37), EAT volume (≥129.14 vs. < 129.14), and TAT volume (≥36.38 vs. < 36.38), with comparisons made using log-rank test. A two-sided *P* < 0.05 was considered statistically significant. The prognostic value of parameters for incident cardiovascular events was evaluated using univariate and multivariate Cox proportional hazards analysis with *P*-values < 0.05 as selection criterion. Results were expressed as hazard ratios (HRs) with 95% confidence intervals (CIs). *P*-value < 0.05 was indicated statistical significance. The receiver operating characteristic (ROC) curve analysis was carried out to evaluate whether perivascular adipose tissue characteristics enhances the predictive performance of the Framingham risk score model for cardiovascular events. The area under the curve (AUC) with 95% CIs was calculated.

## Results

### Clinical characteristics of PCAT, TAT, and EAT

The study involved 220 participants with a mean age of 50.14 years, 65.46% were males and 34.54% were females (Table 1). Comparisons between CKD patients by eGFR tertile revealed that those with lower eGFR had significantly higher levels of systolic blood pressure (SBP), serum phosphorus, and intact parathyroid hormone (iPTH), but lower levels of total cholesterol, triglycerides, HDL and LDL cholesterol, glycated hemoglobin (HbA1c), and white blood cells. Furthermore, attenuation of PCAT and EAT were higher in CKD patients with lower eGFR. However, no significant differences were observed in the volumes of PCAT, TAT, and EAT between CKD patients by tertile of eGFR.

**Table 1:** Baseline characteristics of the whole study population by tertiles of eGFR.

**Parameters**	**Total (*n* = 220)**	**eGFR > 30 (*n* = 71)**	**15 < eGFR ≤ 30 (*n* = 51)**	**eGFR ≤ 15 (*n* = 98)**	***P*-value**
Age (year)	50.14 ± 13.78	51.87 ± 14.95	50.06 ± 14.69	48.94 ± 12.35	0.394
Female sex, *n* (%)	76 (34.54%)	21 (29.58%)	18 (35.29%)	37 (37.75%)	0.540
Scr (μmol/L)	293 (172.25, 533.75)	145 (124.5, 172.5)	2.67 (2.26, 3.01)	577.5 (4.45, 7.999)	**< 0.001**
eGFR (ml/min/1.73m^2^)	18.1 (9.21, 36.24)	42.9 (36.68, 52.31)	20.35 (18.41, 25.00)	8.14 (5.9, 11.4)	**< 0.001**
Proteinuria (g/24 h)	2.08 (1.07, 3.79)	1.77 (0.71, 3.82)	1.58 (0.98, 3.45)	2.14 (1.38, 3.84)	0.339
Hypertension, *n* (%)	176 (80%)	52 (73.24%)	40 (78.43%)	84 (85.71%)	0.128
SBP (mmHg)	139.61 ± 19.21	136.49 ± 17.63	136.76 ± 19.14	143.36 ± 19.85	**0.034**
DBP (mmHg)	84.48 ± 13.61	84.24 ± 12.34	83.76 ± 12.99	85.03 ± 14.86	0.852
Smoking, *n* (%)	71 (32.27%)	24 (33.80%)	14 (27.45%)	33 (33.67%)	0.702
BMI (kg/m^2^)	23.66 ± 3.78	23.69 ± 3.88	23.63 ± 3.93	23.66 ± 3.66	0.997
Total cholesterol (mmol/L)	4.6 (3.8, 5.6)	5.2 (4.4, 5.8)	5 (4.15, 5.7)	4.2 (3.5, 5.1)	**0.001**
Triglycerides (mmol/L)	1.72 (1.28, 2.36)	1.82 (1.32, 2.64)	1.77 (1.29, 2.89)	1.59 (1.24, 2.13)	**0.020**
HDL cholesterol (mmol/L)	1.03 (0.86, 1.24)	1.1 (0.89, 1.31)	1.08 (0.92, 1.36)	0.98 (081, 1.16)	**0.045**
LDL cholesterol (mmol/L)	2.85 (2.31, 3.38)	3.19 (2.64, 3.45)	3.01 (2.35, 3.31)	2.57 (2.05, 3.25)	**0.002**
Phosphorus (mmol/L)	1.41 (1.17, 1.69)	1.17 (1.07, 1.35)	1.32 (1.17, 1.44)	1.71 (1.54, 2.01)	**< 0.001**
Ionic calcium (mmol/L)	2.2 (2.1, 2.3)	2.2 (2.1, 2.31)	2.2 (2.10, 2.29)	2.2 (2.0, 2.3)	0.168
ALP (U/L)	62.5 (51.25, 80.75)	66.5 (53, 83)	58 (48, 73.5)	61.5 (51, 82)	0.172
iPTH (pg/ml)	75.15 (44.67, 169.52)	45.3 (29.4, 57.8)	69.55 (44.9, 99.2)	199.1 (88.7, 324.5)	**< 0.001**
Diabetes, *n* (%)	58 (26.36%)	23 (32.39%)	14 (27.45%)	21 (21.43%)	0.274
HbA1c	5.91 ± 0.92	6.18 ± 1.03	5.8 ± 0.78	5.75 ± 0.86	**0.018**
CRP (mg/L)	2.04 (0.67, 5.47)	1.84 (0.49, 4.27)	1.85 (0.56, 6.99)	2.47 (1.05, 5.51)	0.393
WBC (× 10^9^/L)	7.74 (6.06, 9.38)	8.35 (6.28, 9.65)	8.21 (6.69, 8.98)	7.25 (5.64, 8.83)	**0.011**
Neutrophils (× 10^9^/L)	5.27 (3.97, 6.81)	5.51 (3.98, 8.26)	5.64 (4.54, 6.28)	4.94 (3.82, 6.41)	0.177
Uric acid	447.75 ± 124.63	431.93 ± 116.95	466.33 ± 118.14	450.19 ± 132.88	0.327
Lipid-lowering medication, *n* (%)	64 (29.09%)	21 (29.57%)	14 (27.45%)	29 (29.59%)	0.953
PCAT attenuation (HU)	−78.66 ± 9.71	−82.42 ± 8.56	−78.94 ± 9.03	−75.80 ± 9.98	**< 0.001**
TAT attenuation (HU)	−83.30 ± 5.04	−83.65 ± 4.89	−84.35 ± 4.33	−82.51 ± 5.39	0.083
EAT attenuation (HU)	−80.46 ± 4.85	−81.73 ± 4.53	−80.71 ± 4.67	−79.41 ± 4.97	**0.008**
PCAT volume (cm^3^)	3.33 (2.29, 4.51)	3.33 (2.24, 4.25)	3.56 (2.35, 5.08)	3.33 (2.5, 4.5)	0.527
TAT volume (cm^3^)	35.87 (24.51, 52.77)	37.01 (27.07, 49.79)	39.21 (22.75, 56.11)	32.85 (24.51, 53.68)	0.873
EAT volume (cm^3^)	129.48 (96.30, 182.43)	129.14 (101.06, 174.11)	135.72 (96.36, 180.33)	133.24 (94.45, 186.66)	0.847
CAC score (Agatston)	0 (0, 22.1)	0.2 (0, 23.6)	0 (0, 21.15)	0 (0, 15.3)	0.277
TAC score (Agatston)	0 (0, 173.1)	0 (0, 163.5)	4.4 (0, 482.95)	0 (0, 126.5)	0.529

Body mass index (BMI) was calculated as measured weight in kilograms divided by measured height in square meters. The estimated glomerular filtration rate (eGFR) was calculated by using the simplified MDRD equation: eGFR [ml/min/1.73 m^2^] = 186 × Scr^−1.154^ × (age)^−0.203^ × (0.742, female).

Scr, serum creatinine; DBP, diastolic blood pressure; SBP, systolic blood pressure; HDL, high-density lipoprotein; LDL, low-density lipoprotein; ALP, alkaline phosphatase; iPTH, intact parathyroid hormone; HbA1c;, Glycated hemoglobin; CRP, C-reactive protein; WBC, white blood cell; PCAT, pericoronary adipose tissue; TAT, peri-aortic adipose tissue; EAT, epicardial adipose tissue; CAC, coronary artery calcification; TAC, thoracic aorta calcification.

*P* values < 0.05 are shown in bold.

### Association of PCAT, TAT, or EAT with traditional CVD risk factors

Multivariate linear regression analyses were performed to investigate the relationships between perivascular adipose tissue characteristics and clinical parameters in patients with CKD (Tables 2, 3). The results indicated that smoking status, and serum phosphorus were independently associated with PCAT attenuations. BMI and proteinuria showed significant associations with TAT and EAT attenuation. Additionally, HDL and white blood cell counts were also associated with TAT attenuation, while age, serum creatinine, smoking, and SBP were associated with EAT attenuation. In contrast to attenuation, age and BMI were common significant risk factors associated with the volumes of PCAT, TAT, and EAT. Gender also demonstrated a significant association with TAT volumes, with male patients exhibiting larger volumes of TAT than female patients.

**Table 2:** Clinical factors associated with cardiovascular adipose tissue attenuation in CKD patients.

**Parameters**	**PCAT attenuation (HU)**				**TAT attenuation (HU)**				**EAT attenuation (HU)**			
	**Univariate regression**		**Multivariate regression** ^a^		**Univariate regression**		**Multivariate regression** ^b^		**Univariate regression**		**Multivariate regression** ^c^	
	**Beta**	***P*** **-value**	**Beta**	***P*** **-value**	**Beta**	***P*** **-value**	**Beta**	***P*** **-value**	**Beta**	***P*** **-value**	**Beta**	***P*** **-value**
Age (year)	−0.053	0.432			−0.105	0.121			−0.181	**0.007**	−0.154	**0.018**
Gender (female)	−0.190	**0.005**	−0.100	0.168	0.166	0.104			−0.016	0.813		
Scr (μmol/L)	0.370	**< 0.001**	0.216	0.058	0.115	0.090			0.291	**< 0.001**	0.326	**0.004**
eGFR	−0.318	**< 0.001**			−0.097	0.154			−0.241	**< 0.001**		
Proteinuria (g/24 h)	0.063	0.358			0.246	**< 0.001**	0.313	**< 0.001**	0.179	**0.008**	0.169	**0.012**
BMI (kg/m^2^)	−0.055	0.419			−0.219	**0.001**	−0.196	**0.010**	−0.154	**0.022**	−0.196	**0.002**
Total cholesterol (mmol/L)	−0.038	0.574			0.086	0.206			0.002	0.980		
Triglycerides (mmol/L)	−0.098	0.148			−0.075	0.269			−0.056	0.413		
HDL cholesterol (mmol/L)	0.019	0.785			0.196	**0.004**	0.173	**0.019**	0.072	0.293		
LDL cholesterol (mmol/L)	−0.056	0.411			0.056	0.410			−0.032	0.640		
Diabetes (yes or no)	−0.024	0.724			−0.175	**0.009**	−0.071	0.423	−0.084	0.215		
HbA1c	−0.067	0.376			−0.152	**0.044**	−0.030	0.738	−0.130	0.086		
CRP (mg/L)	−0.018	0.798			−0.124	0.071			−0.039	0.575		
WBC (× 10^9^/L)	−0.1	0.141			−0.167	**0.013**	−0.159	**0.032**	−0.188	**0.005**	−0.196	0.286
Neutrophils (× 10^9^/L)	−0.094	0.165			−0.124	0.067			−0.170	**0.012**	−0.009	0.960
Smoking (yes or no)	0.260	**< 0.001**	0.202	**0.005**	0.022	0.740			0.166	**0.014**	0.142	**0.029**
Hypertension (yes or no)	0.009	0.893			−0.143	**0.034**	−0.027	0.707	−0.052	0.442		
SBP (mmHg)	0.130	0.055			0.097	0.151			0.206	**0.002**	0.182	**0.005**
DBP (mmHg)	−0.081	0.233			0.072	0.289			0.066	0.329		
Phosphorus (mmol/L)	0.344	**< 0.001**	0.194	0.044	0.093	0.171			0.190	**0.005**	0.055	0.522
Ionic calcium (mmol/L)	−0.182	**0.007**	−0.018	0.794	−0.064	0.342			−0.177	**0.009**	−0.084	0.254
ALP (U/L)	−0.128	0.057			−0.212	**0.002**	−0.115	0.114	−0.150	**0.026**	−0.080	0.210
iPTH (pg/ml)	0.263	**< 0.001**	0.055	0.514	0.000	0.991			0.144	**0.035**	0.009	0.923
Uric acid	0.091	0.183			−0.136	**0.047**	−0.057	0.439	−0.008	0.902		
Lipid-lowering medication (yes or no)	0.048	0.517			−0.049	0.509			0.010	0.898		
CAC score	0.026	0.703			−0.053	0.433			−0.020	0.771		
TAC score	0.055	0.417			−0.023	0.732			−0.024	0.722		

Multiple linear regressions (Enter). Variables in multivariable regression models were included based on the *P* value < 0.05. Multivariable regression analysis did not include eGFR which was calculated based on age, sex, and creatinine values.

Beta, standardized coefficient; PCAT, peri-coronary adipose tissue; TAT, peri-aortic adipose tissue; EAT, epicardial adipose tissue; CAC, coronary artery calcification; TAC, thoracic aorta calcification.

*P* values < 0.05 are shown in bold.

^a^R^2^ = 0.218, Durbin–Watson = 2.042; ANOVA F = 7.957, *P* = 0.001.

^b^R^2^ = 0.260, Durbin–Watson = 1.908; ANOVA F = 7.318, *P* < 0.001.

^c^R^2^ = 0.461, Durbin–Watson = 1.893; ANOVA F = 15.400, *P* < 0.001.

**Table 3:** Clinical factors associated with cardiovascular adipose tissue volume in CKD patients.

**Parameters**	**PCAT volume (cm** ^**3**^ **)**				**TAT volume (cm** ^**3**^ **)**				**EAT volume (cm** ^**3**^ **)**			
	**Univariate regression**		**Multivariate regression** ^a^		**Univariate regression**		**Multivariate regression** ^b^		**Univariate regression**		**Multivariate regression** ^c^	
	**Beta**	***P*** **-value**	**Beta**	***P*** **-value**	**Beta**	***P*** **-value**	**Beta**	***P*** **-value**	**Beta**	***P*** **-value**	**Beta**	***P*** **-value**
Age (year)	0.164	**0.015**	0.146	**0.020**	0.376	**< 0.001**	0.305	**< 0.001**	0.363	**< 0.001**	0.323	**< 0.001**
Gender (female)	−0.134	**0.047**	−0.064	0.310	−0.348	**< 0.001**	−0.253	**< 0.001**	−0.207	**0.002**	−0.104	0.051
Scr (μmol/L)	0.012	0.862			−0.006	0.926			−0.015	0.830		
eGFR	−0.043	0.530			0.041	0.546			0.005	0.936		
Proteinuria (g/24 h)	0.087	0.203			0.013	0.849			0.017	0.803		
BMI (kg/m^2^)	0.373	**< 0.001**	0.326	**< 0.001**	0.515	**< 0.001**	0.443	**< 0.001**	0.551	**< 0.001**	0.505	**< 0.001**
Total cholesterol (mmol/L)	0.049	0.470			0.091	0.183			0.038	0.578		
Triglycerides (mmol/L)	0.023	0.734			0.021	0.753			−0.044	0.515		
HDL cholesterol (mmol/L)	−0.089	0.194			−0.122	0.072			−0.124	0.069		
LDL cholesterol (mmol/L)	0.068	0.320			0.118	0.083			0.067	0.324		
Diabetes (yes or no)	0.125	0.065			0.171	**0.011**	−0.031	0.691	0.135	**0.045**	−0.089	0.120
HbA1c	0.069	0.363			0.192	**0.011**	0.009	0.910	0.112	0.139		
CRP (mg/L)	0.077	0.263			0.057	0.405			0.059	0.390		
WBC (× 10^9^/L)	0.037	0.583			0.120	0.075			0.050	0.461		
Neutrophils (× 10^9^/L)	0.020	0.773			0.096	0.156			0.049	0.470		
Smoking (yes or no)	0.118	0.081			0.162	**0.016**	−0.076	0.268	0.113	0.096		
Hypertension (yes or no)	0.177	**0.008**	0.121	0.059	0.118	0.081			0.143	0.034	0.065	0.231
SBP (mmHg)	0.096	0.157			0.014	0.839			0.036	0.594		
DBP (mmHg)	0.023	0.735			−0.077	0.255			0.032	0.634		
Phosphorus (mmol/L)	−0.042	0.532			−0.025	0.718			−0.020	0.771		
Ionic calcium (mmol/L)	−0.017	0.803			−0.116	0.087			−0.101	0.134		
ALP (U/L)	0.116	0.085			0.130	0.055			0.108	0.109		
iPTH (pg/ml)	0.067	0.327			0.087	0.205			0.071	0.299		
Uric acid	0.136	0.046			0.180	**0.008**	0.077	0.212	0.110	0.108		
Lipid-lowering medication (yes or no)	0.052	0.482			0.089	0.228			0.048	0.520		
CAC score	0.061	0.369			0.139	**0.040**	0.017	0.791	0.164	**0.015**	0.075	0.191
TAC score	0.006	0.934			0.207	**0.002**	0.069	0.288	0.183	**0.007**	0.034	0.549

Multiple linear regressions (Enter). Variables in multivariable regression models were included based on the *P* value < 0.05.

Beta, standardized coefficient; PCAT, peri-coronary adipose tissue; TAT, peri-aortic adipose tissue; EAT, epicardial adipose tissue; CAC, coronary artery calcification; TAC, thoracic aorta calcification.

*P* values < 0.05 are shown in bold.

^a^R^2^ = 0.167, Durbin–Watson = 2.043; ANOVA F = 7.077, *P* < 0.001.

^b^R^2^ = 0.445, Durbin–Watson = 1.992; ANOVA F = 27.899, *P* < 0.001.

^c^R^2^ = 0.453, Durbin–Watson = 1.846; ANOVA F = 36.858, *P* < 0.001.

### Relationships of PCAT, TAT, or EAT characteristics with cardiovascular outcomes

**Figure 2:**
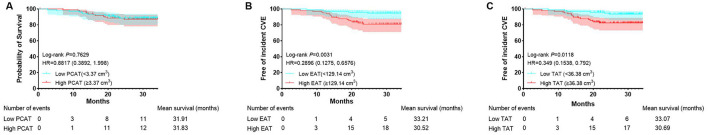
Kaplan–Meier curves stratified by volumes of PCAT, EAT, and TAT. Kaplan–Meier curves stratified by PCAT volume **(A)**. Kaplan–Meier curves stratified by EAT volume **(B)**. Kaplan–Meier curves stratified by TAT volume **(C)**. CVE, cardiovascular events.

After excluding 23 patients lost to follow-up, 197 patients were included in the follow-up study (Figure 1). The mean follow-up duration was 26.56 months, ranging from 2.67 to 34.20 months. During this period, 23 incident cardiovascular events occurred, including four cardiovascular-related deaths, four coronary revascularization procedures, and 16 hospitalizations due to heart failure. There were three patients died from non-cardiovascular causes, including COVID19-related complications. Patients with cardiovascular outcomes were found to be older, with higher rates of hypertension and smoking occurrence, higher proteinuria and SBP, greater BMI, higher CAC and TAC scores, and larger volumes of EAT and TAT compared to CKD patients without cardiovascular outcomes (Supplementary Table S1). Kaplan–Meier survival curves showed that patients with high EAT volume (≥129.14 cm^3^) or TAT volume (≥36.38 cm^3^) suffered significantly more incident cardiovascular events than patients with low volumes (*P* < 0.05; Figure 2).

Furthermore, Cox regression models were utilized to assess the impact of perivascular adipose tissue characteristics on cardiovascular outcomes (Supplementary Table S2). EAT volume emerged as an independent risk factor for incident cardiovascular events after multivariable regression (HR = 1.009, *P* = 0.01, Table 4). However, during the short-term follow-up, neither the volumes nor the attenuations of TAT and PCAT were found to be correlated with cardiovascular risk.

**Table 4:** Cox regression analysis of perivascular adipose tissue for incident cardiovascular events.

**Parameters**	**Model 1**		**Model 2**	
	**HR (95% CI)**	***P*** **-value**	**HR (95% CI)**	***P*** **-value**
PCAT volume	1.271 (1.015, 1.592)	**0.037**	1.076 (0.833, 1.391)	0.575
EAT volume	1.009 (1.005, 1.014)	**< 0.001**	1.009 (1.002, 1.016)	**0.010**
TAT volume	1.016 (1.001, 1.031)	**0.032**	1.007 (0.985, 1.030)	0.516
PCAT attenuation	0.994 (0.953, 1.036)	0.767		
EAT attenuation	0.944 (0.867, 1.028)	0.185		
TAT attenuation	1.008 (0.929, 1.093)	0.852		

Model 1: unadjusted. Model 2: adjusted for age, smoking, systolic blood pressure (SBP), body mass index (BMI), and coronary artery calcium (CAC) score. Multivariate regression was performed using the enter method. *P* values < 0.05 are shown in bold.

**Figure 3:**
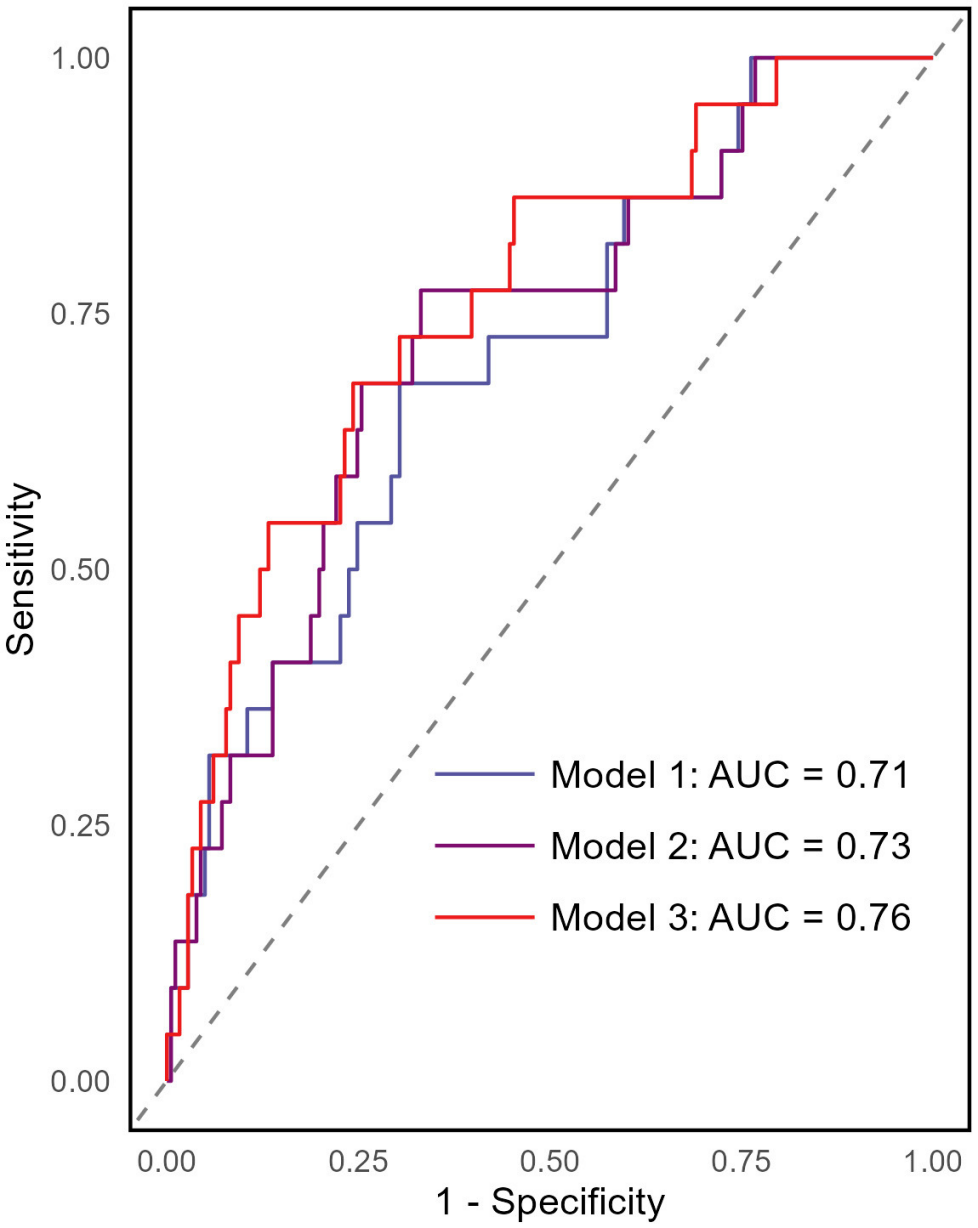
Receiver operating characteristics (ROC) curves for different prediction models of incident cardiovascular events. The area under the ROC curve (AUC) was used to assess the predictive performance of the models. The analysis included three models: Model 1 included only the Framingham risk score (AUC = 0.71, 95% CI 0.59–0.82). Model 2 incorporated the Framingham risk score and CAC score (AUC = 0.73, 95% CI 0.62–0.84). Model 3 included EAT volume in addition to Model 2, leading to an AUC of 0.76 (95% CI 0.66–0.87).

To assess whether EAT volume improves the predictive performance of the Framingham risk score model for cardiovascular events in non-dialysis CKD patients, ROC analysis with AUC was conducted. The analysis compared different models: model 1 included only the Framingham risk score (AUC 0.71, 95% CI 0.59–0.82); model 2 added the CAC score (AUC 0.73, 95% CI 0.62–0.84); and model 3 included EAT volume (AUC 0.76, 95% CI 0.66–0.87; Figure 3). These results indicate that EAT volume provides an incremental improvement in the model's predictive ability.

## Discussion

This observational study evaluated the relationships between perivascular adipose tissue (PCAT, TAT, and EAT) volumes/attenuations and incident cardiovascular outcomes in non-dialysis CKD patients. Our analysis found that age and BMI were independently associated with these tissues' volumes. Smoking and serum phosphorus levels correlated with PCAT attenuation, whereas proteinuria, BMI, HDL cholesterol, and white blood cell counts were linked to TAT attenuation. EAT attenuations was associated with age, serum creatinine, proteinuria, BMI, smoking, and systolic blood pressure. Importantly, high baseline volumes of EAT and TAT were linked to higher incident cardiovascular event rates, with EAT volume emerging as an independent predictor of such outcome in multivariate analysis. EAT volume also enhanced the Framingham risk score's predictive value. These findings indicate that EAT volume serves as potential biomarker for cardiovascular risk assessment in non-dialysis CKD patients.

One novelty of this study is its identification of EAT volume's significant and independent predictive value for incident cardiovascular outcomes in non-dialysis CKD patients. EAT, primarily acting as perivascular adipose tissue for the coronary arteries, plays a pivotal role in the development of various CVDs, such as coronary artery disease, heart failure, and atrial fibrillation (5, 16, 17). A recent systematic review and meta-analysis by Chong et al. (3) provides evidence that EAT volume was associated with adverse cardiovascular outcomes, including cardiac death, myocardial infarction, coronary revascularization, and atrial fibrillation. However, whether EAT has a direct link to incident CVDs remains debated (3). Recent studies have identified EAT as a cardiovascular risk factor, particularly among patients with end-stage renal disease, which did not assess EAT in patients with and without prior cardiovascular pathology (18). Patients with CKD were found to have significantly higher EAT volumes than individuals without CKD, and this increased EAT volume was associated with the presence of high-risk plaque (19). Despite the relatively short follow-up period of our study, EAT characteristics showed significant associations with the prognosis of incident cardiovascular event. Previous research indicated that PCAT is associated with increased cardiovascular risk (20–22), likely due to its role in secreting proinflammatory cytokines and inducing inflammation in the adjacent coronary artery under pathological states, thereby promoting arteriosclerosis, atherosclerosis, and other CVD events (23–26). However, in our study of non-dialysis CKD patients, both PCAT and TAT were not significantly associated with incident cardiovascular outcomes after adjusting risk factors.

Identifying EAT characteristics as biomarkers for cardiovascular risk has significant clinical implications. Our findings suggest that incorporating EAT assessment into routine cardiovascular risk evaluation could enhance risk prediction and improve the detection of high-risk non-dialysis CKD patients. This improved risk stratification may allow timely interventions to prevent or mitigate cardiovascular events. Furthermore, EAT assessment offers several advantages as a novel biomarker. CT scans are relatively non-invasive and can be efficiently quantified from images, with accuracy and speed now enhanced by artificial intelligence technology (27, 28). Moreover, EAT provides insights beyond traditional cardiovascular risk factors. It reflects local inflammatory and metabolic processes within the perivascular environment, which are closely tied to the development and progression of atherosclerosis and other vascular diseases (29). This unique perspective may help identify high-risk individuals who might otherwise be overlooked by conventional risk assessment tools like the Framingham risk score.

Under kidney dysfunction and other factors, peri-vascular adipose tissue can adopt detrimental properties and actively contribute to CVD pathophysiology (30). Compared to individuals with normal renal function, patients with CKD have significantly increased volumes of cardiovascular adipose tissue (7, 19). In our cohort, EAT attenuation was higher in CKD patients with lower eGFR, indicating altered EAT characteristics in renal failure. Also, EAT volume was greater in non-dialysis CKD patients with cardiovascular outcome than in those without. Increased perivascular adipose tissue volume is correlated with the secretion of inflammatory adipocytokines, linking it to a range of diseases. Alterations in EAT can lead to macrophage infiltration and inflammation, which may influence vascular function and contribute to the development and progression of CVD (16, 31, 32). Animal studies further support the pathological role of adipokines from perivascular adipose tissue including EAT in promoting vascular dysfunction, atherosclerosis, and cardiac remodeling (33, 34). EAT displays high rates of lipogenesis and lipolysis and is proposed to serve as local fat storage depot (29, 35). Its dysfunction is interrelated with inflammation and oxidative stress, contributing to endothelial dysfunction in conditions like obesity and hypertension (36, 37). These findings suggest that EAT may be a potential therapeutic target for the prevention and treatment of CVD, particularly in patients with CKD.

This study has several strengths and limitations. A key strength is that it is the first to simultaneously measure PCAT, TAT, and EAT using non-enhanced CT scanning in non-dialysis patients with CKD. Our findings support the clinical applicability of non-enhanced CT scanning for measuring cardiovascular adipose tissue in patients with renal failure. In addition, both cardiovascular adipose tissue attenuation and volume, and vascular calcification were quantified, allowing for a thorough evaluation of their associations with incident cardiovascular outcomes. However, the study's limitations include its small sample size, short-term follow-up, and single-center design. Also, the inclusion criteria limited the population to CKD patients with available CT scans, making it challenging to compare findings with the general population or those without CKD. Longitudinal studies have suggested that the predictive value of EAT thickness for adverse cardiovascular outcomes increases with longer follow-up (18, 19), reinforcing the need to evaluate EAT volume in larger cohort of CKD cohorts and with long-term follow-up to determine its prognostic significance.

## Conclusions

This study revealed that EAT volume has high predictive value for incident cardiovascular outcome in non-dialysis CKD patients. These findings underscore the importance of monitoring EAT in patients with non-dialysis CKD. Given that CKD is characterized by pronounced and rapidly progressing CVD, understanding epicardial adipose tissue's role is essential for elucidating the underlying pathophysiology and improving cardiovascular health management strategies in this population. Implementing routine assessments of epicardial adipose tissue characteristics could ultimately guide therapeutic decisions and enhance patient care.

## Data Availability

The raw data supporting the conclusions of this article will be made available by the authors, without undue reservation.
