# Supplementary material for: The impact of perivascular adipose tissue characteristics on incident cardiovascular events in non-dialysis chronic kidney disease patients
Source: Front Med (Lausanne). 2025 Jun 20;12:1547007. doi: 10.3389/fmed.2025.1547007 (PMC12226465; doi:10.3389/fmed.2025.1547007)
Supplement: Supplementary file 1 [file Data_Sheet_1.docx]

Supplementary data


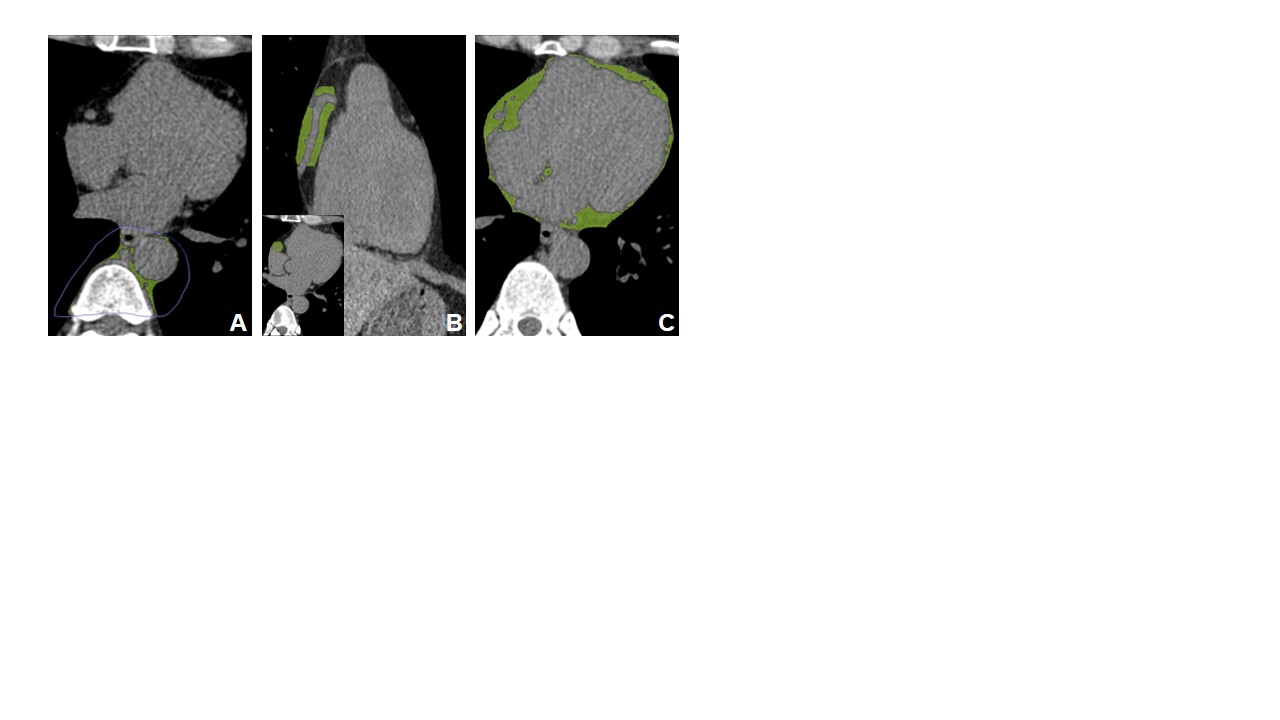


**Figure S1. CT reveals thoracic peri-aortic, pericoronary, and pericardial adipose tissue.** (A) Axial view of thoracic peri-aortic adipose tissue is defined by the fat surrounding the thoracic aorta and is highlighted in green. (B) Axial and corresponding cross-sectional view of pericoronary adipose tissue is defined by the fat surrounding the proximal right coronary artery and highlighted in green. (C) The axial view of epicardial adipose tissue is defined as the fat between the heart and the pericardium and is highlighted in green.

**Table S1: Baseline characteristics of follow-up population with and without cardiovascular outcome**

| **Parameters** | **Total (n=197)** | **With CVE (n=23)** | **Without CVE (n=174)** | ***P* value** |
| --- | --- | --- | --- | --- |
| Age (year) | 50.14±13.78 | 56.78±13.79 | 49.55±13.54 | **0.025** |
| Female sex, n (%) | 70 (35.53 %) | 6 (26.09 %) | 64 (36.78 %) | 0.314 |
| Scr (umol/L) | 284 (163, 520) | 338 (225, 552) | 278 (160, 501) | 0.284 |
| eGFR (ml/min/1.73m^2^) | 18.85 (9.85, 38.48) | 13.2 (10.39, 23.94) | 20.24 (10.33, 40.27) | 0.167 |
| Proteinuria (g/24 h) | 1.84 (0.94, 3.59) | 3.51 (1.66, 3.82) | 1.73 (0.97, 3.17) | **0.041** |
| Hypertension, n (%) | 156 (79.19 %) | 22 (95.65 %) | 134 (77.01 %) | 0.038 |
| SBP (mmHg) | 139.61±19.21 | 150.83±20.89 | 137.02±18.17 | **0.005** |
| DBP (mmHg) | 84.48±13.61 | 86.35±14.42 | 84.092±12.77 | 0.481 |
| Smoking, n (%) | 64 (32.49 %) | 12 (52.17 %) | 52 (29.88 %) | **0.032** |
| BMI (kg/m^2^) | 23.66 ± 3.78 | 25.47±4.28 | 23.31±3.60 | **0.028** |
| Total cholesterol (mmol/L) | 4.6 (3.8, 5.6) | 4.6 (3.4, 5.7) | 4.6 (3.8, 5.4) | 0.882 |
| Triglycerides (mmol/L) | 1.69 (1.28, 2.35) | 1.54 (1.39, 1.76) | 1.75 (1.28, 2.37) | 0.154 |
| HDL (mmol/L) | 1.03 (0.86,1.24) | 0.99 (0.87, 1.27) | 1.04 (0.86, 1.24) | 0.729 |
| LDL (mmol/L) | 2.81 (2.33, 3.37) | 2.79 (1.99, 3.69) | 2.85 (2.35, 3.36) | 0.860 |
| Phosphorus (mmol/L) | 1.38 (1.17, 1.68) | 1.46 (1.27, 1.58) | 1.36 (1.16, 1.69) | 0.689 |
| Ionic calcium (mmol/L) | 2.2 (2.1, 2.3) | 2.2 (2.1, 2.3) | 2.2 (2.1, 2.3) | 0.970 |
| ALP (U/L) | 62 (51,78) | 58 (45, 81) | 63 (52, 78) | 0.272 |
| iPTH (pg/ml) | 74.4 (44.7, 157) | 110 (63, 168.20) | 73.5 (44.6, 147.8) | 0.098 |
| Diabetes, n (%) | 52 (26.39 %) | 9 (39.13 %) | 43 (24.71 %) | 0.140 |
| CRP (mg/L) | 2.04 (0.64, 5.54) | 5.27 (0.6, 6.49) | 2.01 (0.72, 4.55) | 0.169 |
| WBC (x10^9/L) | 7.67 (5.99, 9.3) | 8.03 (6.86, 8.70) | 7.52 (5.95, 9.32) | 0.706 |
| Neutrophils (x10^9/L) | 5.16 (3.93, 6.48) | 5.28 (4.84, 5.95) | 5.16 (3.77, 6.42) | 0.585 |
| Anti-diabetic medication, n (%) | 39 (19.80 %) | 6 (26.09 %) | 33 (18.97 %) | 0.376 |
| Lipid-lowering medication, n (%) | 57 (28.93 %) | 7 (30.43 %) | 50 (28.73 %) | 0.667 |
| PCAT attenuation (HU) | -78.66±9.71 | -79.30±9.04 | -78.52±9.86 | 0.703 |
| TAT attenuation (HU) | -83.30±5.04 | -83.13±4.09 | -83.40±5.21 | 0.779 |
| EAT attenuation (HU) | -80.46±4.85 | -81.65±6.32 | -80.24±4.61 | 0.308 |
| PCAT volume (cm3) | 3.37 (2.35, 4.56) | 4.21 (2.91, 5.88) | 3.37 (2.33, 4.49) | 0.098 |
| TAT volume (cm3) | 36.38 (24.5, 52.11) | 49.54 (37.34, 57.55) | 34.6 (24.22, 49.97) | **0.006** |
| EAT volume (cm3) | 129.14 (95.99, 179.32) | 179.26 (132.60, 234.63) | 126.86 (94.45, 174.11) | **0.001** |
| CAC score (Agatston) | 0 (0, 21.7) | 54.9 (0, 217.4) | 0 (0, 14.40) | **0.003** |
| TAC score (Agatston) | 0 (0, 187) | 209.8 (1.15, 1349.8) | 0 (0, 136.7) | **0.006** |

Scr, serum creatinine; eGFR, estimated glomerular filtration rate; DBP, diastolic blood pressure; SBP, systolic blood pressure; BMI, Body mass index; HDL, high-density lipoprotein; LDL, low-density lipoprotein; ALP, alkaline phosphatase; iPTH, intact parathyroid hormone; HbA1c, Glycated hemoglobin; CRP, C-reactive protein; WBC, white blood cell; PCAT, pericoronary adipose tissue; TAT, peri-aortic adipose tissue; EAT, epicardial adipose tissue; CAC, coronary artery calcification; TAC, thoracic aorta calcification.

**Table S2. Cox regression of risk factors associated with cardiovascular outcomes in CKD patients**

| **Parameters** | **Cardiovascular outcomes** | | | | | | | |
| --- | --- | --- | --- | --- | --- | --- | --- | --- |
|  | Univariate regression | | Multivariate regression^a^ | | Multivariate regression^b^ | | Multivariate regression^c^ | |
|  | HR (95% CI) | *P* | HR (95% CI) | *P* | HR (95% CI) | *P* | HR (95% CI) | *P* |
| Age (year) | 1.039 (1.007, 1.072) | **0.018** | 1.034 (1, 1.069) | 0.053 | 1.019 (0.982, 1.058) | 0.315 | 1.031 (0.994, 1.068) | 0.100 |
| Gender (female) | 0.603 (0.238, 1.531) | 0.287 |  |  |  |  |  |  |
| Scr (umol/L) | 1 (0.999, 1.002) | 0.681 |  |  |  |  |  |  |
| eGFR ((ml/min/1.73m^2^)) | 0.981 (0.955, 1.008) | 0.159 |  |  |  |  |  |  |
| Proteinuria (g/24h) | 1.032 (0.934, 1.141) | 0.531 |  |  |  |  |  |  |
| BMI (kg/m^2^) | 1.169 (1.047, 1.306) | **0.006** | 1.167 (1.028, 1.326) | 0.017 | 1.069 (0.920, 1.242) | 0.385 | 1.153 (1.002, 1.326) | **0.046** |
| total cholesterol (mmol/L) | 0.955 (0.729, 1.252) | 0.739 |  |  |  |  |  |  |
| Triglycerides (mmol/L) | 0.680 (0.396, 1.170) | 0.164 |  |  |  |  |  |  |
| HDL cholesterol (mmol/L) | 0.984 (0.317, 3.058) | 0.978 |  |  |  |  |  |  |
| LDL cholesterol (mmol/L) | 0.954 (0.635, 1.432) | 0.820 |  |  |  |  |  |  |
| Diabetes (yes or no) | 1.901 (0.823, 4.393) | 0.133 |  |  |  |  |  |  |
| CRP (mg/L) | 1.004 (0.977, 1.032) | 0.775 |  |  |  |  |  |  |
| Smoking (yes or no) | 2.439 (1.075, 5.530) | **0.033** | 2.120 (0.896, 5.019) | 0.087 | 2.369 (0.996, 5.638) | 0.051 | 1.979 (0.854, 4.584) | 0.111 |
| Hypertension (yes or no) | 6.209 (0.837, 46.068) | 0.074 |  |  |  |  |  |  |
| SBP (mmHg) | 1.033 (1.013, 1.054) | **0.001** | 1.026 (1.004, 1.049) | **0.023** | 1.032 (1.008, 1.056) | **0.007** | 1.029 (1.005, 1.052) | **0.015** |
| DBP (mmHg) | 1.013 (0.982, 1.045) | 0.427 |  |  |  |  |  |  |
| Phosphorus (mmol/L) | 0.857 (0.307, 2.388) | 0.767 |  |  |  |  |  |  |
| Ionic calcium (mmol/L) | 1.217 (0.144, 10.298) | 0.857 |  |  |  |  |  |  |
| ALP (U/L) | 0.991 (0.972, 1.009) | 0.331 |  |  |  |  |  |  |
| iPTH (pg/ml) | 1 (0.997, 1.003) | 0.899 |  |  |  |  |  |  |
| Lipid-lowering medication (yes or no) | 1.202 (0.451, 3.202) | 0.713 |  |  |  |  |  |  |
| CAC score | 1.004 (1.002, 1.005) | **<0.001** | 1.003 (1.002, 1.005) | **<0.001** | 1.003 (1.002, 1.005) | **<0.001** | 1.003 (1.002,1.005) | **<0.001** |
| TAC score | 1 (1, 1) | 0.170 |  |  |  |  |  |  |
| PCAT volume | 1.271 (1.015, 1.592) | **0.037** | 1.076 (0.833, 1.391) | 0.575 |  |  |  |  |
| EAT volume | 1.009 (1.005, 1.014) | **<0.001** |  |  | 1.009 (1.002, 1.016) | **0.010** |  |  |
| TAT volume | 1.016 (1.001, 1.031) | **0.032** |  |  |  |  | 1.007 (0.985, 1.030) | 0.516 |
| PCAT attenuation | 0.994 (0.953, 1.036) | 0.767 |  |  |  |  |  |  |
| EAT attenuation | 0.944 (0.867, 1.028) | 0.185 |  |  |  |  |  |  |
| TAT attenuation | 1.008 (0.929, 1.093) | 0.852 |  |  |  |  |  |  |

Cox regressions (Enter). Variables in multivariate regression models were included based on the *P* value < 0.05. aHR: hazard ratios; CI: confidence intervals; PCAT, peri-coronary adipose tissue; TAT, peri-aortic adipose tissue; EAT, epicardial adipose tissue; CAC, coronary artery calcification; TAC, thoracic aorta calcification. *P* value < 0.05 was indicated in bold.
